# Supplementary material for: Regulation of Amylose Content by Single Mutations at an Active Site in the Wx-B1 Gene in a Tetraploid Wheat Mutant
Source: Int J Mol Sci. 2022 Jul 29;23(15):8432. doi: 10.3390/ijms23158432 (PMC9368913; doi:10.3390/ijms23158432)
Supplement: Supplementary file 1 [file ijms-23-08432-s001.zip › ijms-1794241-supplementary.pdf]

Supplementary Materials:

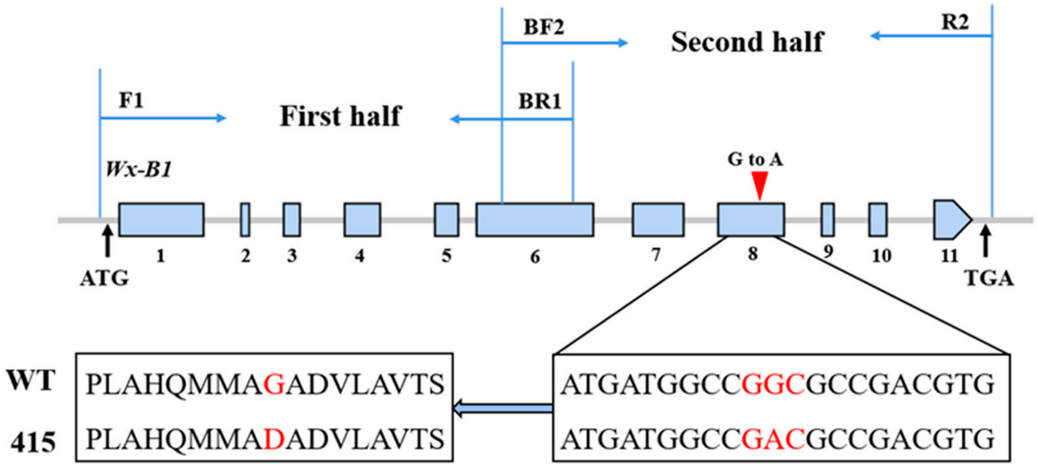

Figure S1. Analysis of *Wx-B1* gene sequences in wheat parental WT and mutant M3-415 lines.

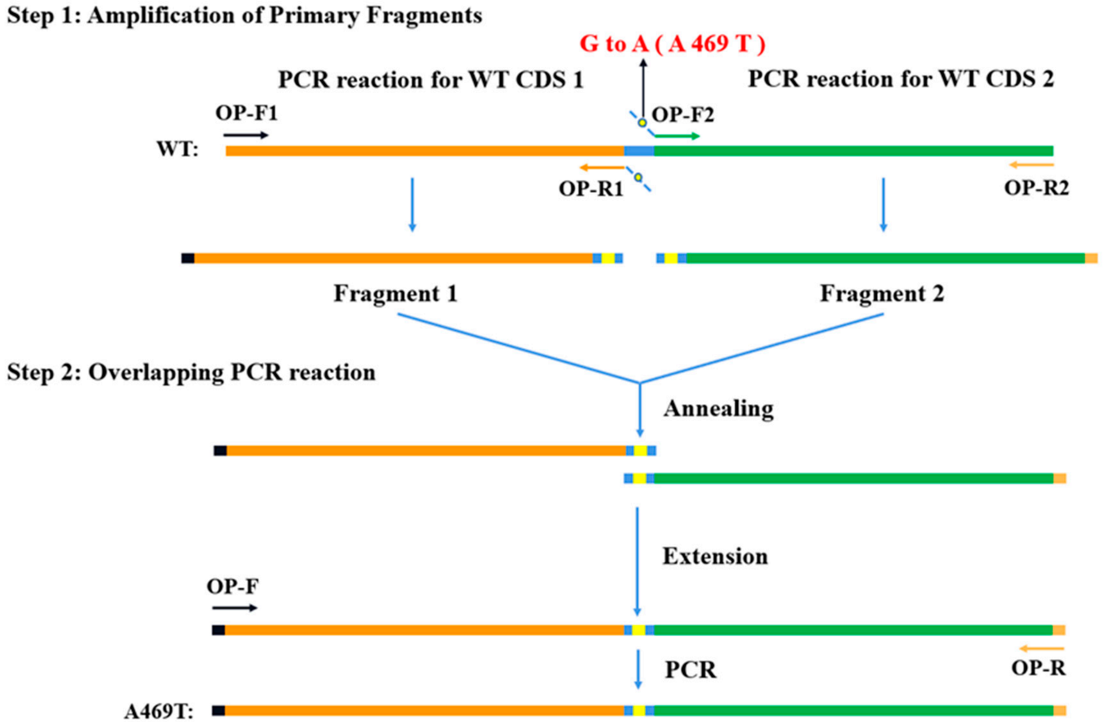

Figure S2. Schematic diagram of the PCR site-directed mutagenesis of the *Wx-B1* gene conducted using the overlap extension method. In step 1, touchdown PCR was used to generate PCR fragments that contained a 20-bp *Wx-B1* sequence overlap (blue portion of primers OP-F2 and OP-R1, yellow portion of site-directed mutagenesis position) at the 3′ end of Fragment 1 and 5′ end of Fragment 2. In step 2, the two fragments were used to generate a full length *Wx-B1* coding sequence via overlap PCR.

Table S1. Primers used in this study.

Primers	Sequence (5′-3′)	Tm (°C)	Description
F1	ATGGCGGCTCTGGTCACGT	60	*Wx-B1* amplification
BR1	ACGATGCCGGTGATGCCC	59	
BF2	GTTCTGCATCCACAACATCTCGTAT	60	*Wx-B1* amplification
R2	TCAGGGAGCGGCGACGTT	60	
OP-F	ATGGCGGCTCTGGTCACG	60	TB-1 amplification
OP-R	TCAGGGAGCGGCGACGTT	60	
OP-F1	ATGGCGGCTCTGGTCACGTCGCAG	61	TB-1 amplification
OP-R1	CGCCGGTCATCATCTGGTGA	60	
OP-F2	TCACCAGATGATGACCGGCG	59	TB-1 amplification
OP-R2	TCAGGGAGCGGCGACGTTCTCCATG	60	
TY-F	ATCGGATCCGAATTCGAGCTCATGGCGGCTCTGGTCACG	62	*Wx-B1* amplification
TY-R	CTCGAGTGCGGCCGCAAGCTTTCAGGGAGCGGCGACGTT	64	
GAPDH-F	AACTGTTCATGCCATCACTGCCAC	58	Internal control gene
GAPDH-R	AGGACATACCAGTGAGCTTGCCAT	60	
Actin-F	GTTCCAATCTATAAGGGATACACGC	60	Internal control gene
Actin-R	GAACCTCCACTGAGAACAACATTACC	60	
Wx-F	CTCAACAAACAACCCATACTTCTCC	60	*Wx-B1* amplification
Wx-R	CGCTACCTTGGCCGTCCTA	58	*Wx-B1* amplification
